# The outcome of experimentally induced inclusion body hepatitis (IBH) by fowl aviadenoviruses (FAdVs) is crucially influenced by the genetic background of the host

**DOI:** 10.1186/s13567-016-0350-0

**Published:** 2016-06-29

**Authors:** Miguel Matos, Beatrice Grafl, Dieter Liebhart, Michael Hess

**Affiliations:** Clinic for Poultry and Fish Medicine, Department for Farm Animals and Veterinary Public Health, University of Veterinary Medicine Vienna, Veterinaerplatz 1, 1210 Vienna, Austria; Christian Doppler Laboratory for Innovative Poultry Vaccines (IPOV), University of Veterinary Medicine Vienna, Veterinaerplatz 1, 1210 Vienna, Austria

## Abstract

In the present study, inclusion body hepatitis (IBH) was experimentally induced by oral inoculation of two groups of specific pathogen-free (SPF) broilers and two groups of SPF layers at day-old with either a fowl aviadenovirus (FAdV)-D or a FAdV-E strain. A substantial variation in the degree of susceptibility was observed with mortalities of 100 and 96% in the FAdV-E and D infected SPF broiler groups, respectively, whereas in the groups of infected SPF layers mortalities of only 20 and 8% were noticed. Significant changes in clinical chemistry analytes of all infected birds together with histopathological lesions indicated impairment of liver and pancreas integrity and functions. Furthermore, significantly lower blood glucose concentrations were recorded at peak of infection in both inoculated SPF broiler groups, in comparison to the control group, corresponding to a hypoglycaemic status. High viral loads were determined in liver and pancreas of SPF broilers already at 4 days post-infection (dpi), in comparison to SPF layers, indicating a somewhat faster viral replication in the target organs. Overall, highest values were noticed in the pancreas of SPF broilers independent of the virus used for infection. The actual study provides new insights into the pathogenesis of IBH, a disease evolving to a metabolic disorder, to which SPF broilers were highly susceptible. Hence, this is the first study to report a significant higher susceptibility of SPF broiler chickens to experimentally induced IBH in direct comparison to SPF layers.

**Electronic supplementary material:**

The online version of this article (doi:10.1186/s13567-016-0350-0) contains supplementary material, which is available to authorized users.

## Introduction

Fowl aviadenoviruses (FAdVs) belong to the genus *Aviadenovirus* within the family *Adenoviridae*, being further divided into five species designated FAdV-A to E [1]. Throughout the years, many reports established a causality between strains from species FAdV-A, FAdV-C and FAdV-D together with FAdV-E with specific diseases in chickens, such as adenoviral gizzard erosion (AGE), hydropericardium hepatitis syndrome (HHS) and inclusion body hepatitis (IBH), respectively [2].

In the last 10 years IBH outbreaks have been reported in different geographic regions emphasizing the wide distribution of FAdVs throughout the world [3–8]. In the field, IBH has been reported essentially from commercial broiler flocks (meat-producing chickens), being responsible for serious economic losses due to increased mortality combined with reduced performance within flocks [2]. However, experimental in vivo studies were predominantly conducted in specific pathogen-free (SPF) white leghorn layers (egg-producing chickens), which are the experimental model for infection studies.

In a recent study we were able to demonstrate the influence of virulent FAdV-D and E field strains on different enzyme systems and metabolite concentrations in the plasma of orally inoculated day-old SPF layer chickens due to the infection of liver and pancreas as target organs [9]. Consequently, it can be hypothesized that hosts with different metabolic activities vary in their susceptibilities towards infection. Therefore, the aim of the present study was to characterize and compare the susceptibility of SPF broiler and SPF layer chickens to experimentally induced IBH by FAdV-D and E field strains.

## Materials and methods

### Viruses

The FAdV strains used in the present study—08/18 926 and 13/18 153—were isolated from liver samples of broilers during recent IBH outbreaks in Europe and they were genotyped as belonging to species FAdV-D and E, respectively [4, 8]. The viruses were plaque purified three times and propagated in primary chicken embryo liver (CEL) cell cultures as described elsewhere [10]. The titers were determined according to the method of end point titration [11] and a titer of 10^7^ median tissue culture infective dose (TCID_50_) per mL was used to infect the birds. A polymerase chain reaction (PCR) and a reverse transcription-PCR were performed to confirm the absence of contaminations by chicken anaemia virus and avian reovirus, respectively. The strains’ pathogenicity was characterized in vivo by inoculating SPF white leghorn chickens at day-old [9].

### Animal trial

Embryonated SPF broiler eggs (Animal Health Service, Deventer, The Netherlands) and SPF layer eggs (VALO, Lohmann Tierzucht GmbH, Cuxhaven, Germany) were incubated at our facilities. After hatch, the chicks were individually tagged subcutaneously (Swiftack, Heartland Animal Health Inc., Fair Play, USA) and divided in six groups: three groups of 27 SPF broiler chicks (groups B0–2) and three groups of 20 SPF layer chicks (groups L0–2). The groups were housed separately in isolator units (Montair Andersen bv, HM 1500, Sevenum, Netherlands) under negative pressure, where feed and water were available ad libitum throughout the animal experiment. At first day of life, the body weight of all birds was measured and birds from groups L1 and B1, and from groups L2 and B2 were orally inoculated with 0.5 mL of the 13/18 153 and the 08/18 926 strains, respectively, while birds from groups L0 and B0 were left uninoculated (Table 1). All birds were daily monitored and an individual score was given based on clinical signs: 0—active with no adverse clinical signs; 1—slightly weak with dropped wings; 2—depressed with swollen crops; 3—weak, apathetic, with ruffled feathers and reluctant to move; 4—apathetic, unable to move or stand, breathing intensely with eyes closed. Euthanasia was applied to birds clinically rated with the highest score. The body weight of all birds was measured at 4, 7, 10, 14 and 21 days post-infection (dpi). Furthermore, at 4 dpi four randomly selected birds of each group were blood sampled, euthanized and necropsied (Table 1). The same procedure was performed at 7, 10, 14 and 21 dpi in groups L1, L2 and L0, whereas in groups B1 and B2 blood was collected from five birds in poor condition, prior euthanasia and subsequent necropsy, between 5 and 7 dpi. In group B0 five and eight randomly selected birds were sampled, euthanized and necropsied at 7, 10 and 14 or 21 dpi, respectively.

**Table 1:** **Experimental design and mortality of birds after oral inoculation with FAdV isolates**

Group	FAdV strain (species)	Chicken type	Sampling scheme and mortality on the following days after inoculation										No. of birds
				4	5	6	7	8	9	10	14	21	
L1	13/18 153 (FAdV-E)	Layer	Killed birds	4	_	_	3	_	_	4	4	2	20
			Dead birds^a^	_^b^	_	1	1	1	_	_	_	_	
B1	13/18 153 (FAdV-E)	Broiler	Killed birds	4	_	_	_	_	_	_	_	_	27
			Dead birds	_	13	10	_	_	_	_	_	_	
L2	08/18 926 (FAdV-D)	Layer	Killed birds	4	_	_	4	_	_	4	4	3	20
			Dead birds	_	_	_	_	_	1	_	_	_	
B2	08/18 926 (FAdV-D)	Broiler	Killed birds	4	_	_	1	_	_	_	_	_	27
			Dead birds	_	4	16	2	_	_	_	_	_	
L0	_^c^	Layer	Killed birds	4	_	_	4	_	_	4	4	4	20
			Dead birds	_	_	_	_	_	_	_	_	_	
B0	_^c^	Broiler	Killed birds	4	_	_	5	_	5	_	5	8	27
			Dead birds	_	_	_	_	_	_	_	_	_	

Two groups of SPF broilers (B1–2) and two groups of SPF layers (L1–2) were inoculated orally at day-old with either a FAdV-D or -E strain, whereas one group SPF broilers (B0) and one group of SPF layers (L0) were kept uninfected. Birds were routinely euthanized and sampled at 4, 7, 10, 14 and 21 dpi. In groups B1 and B2, five birds with severe clinical signs were sampled between 5 and 7 dpi.

^a^Birds found dead or had to be euthanized due to poor condition.

^b^Not applicable.

^c^Control group.

The animal trial was discussed and approved by the institutional ethics committee and the national authority according to §26 of the Law for Animal Experiments, Tierversuchsgesetz 2012—TVG 2012, license number: bmwf GZ 68.205/0041-WF/II/3b/2014.

### Clinical chemistry

Preceding euthanasia, blood was collected from the jugular vein of the birds into heparin tubes (VACUETTE^®^, Greiner Bio-One, Kremsmünster, Austria) and centrifuged at 1780 rcf for 12 min. Plasma was then separated and the values of the following clinical chemistry analytes were investigated by a fully selective clinical chemistry analyzer (Cobas 501c^®^, Roche Diagnostics, Vienna, Austria): total protein, albumin, aspartate aminotransferase (AST), glutamate dehydrogenase (GLDH), bile acids, uric acid, lipase and glucose. All assays were applied according to manufacturer’s recommendations (Additional file 1). The quality control was performed by analysing two levels of control material before each run.

### Post-mortem examination

All euthanized and dead birds throughout the experiment were examined by necropsy and gross lesions in liver, pancreas, bursa of Fabricius and kidneys were recorded. Specimens of these organs were further collected for histopathological investigations. In addition, samples of liver and pancreas were collected to determine the viral load by real-time PCR.

### Histopathology

Samples of liver, pancreas, bursa of Fabricius and kidney were fixed in 4% neutral buffered formalin and embedded in paraffin blocks. Tissue sections with 4 μm of thickness were prepared using a microtome (Microm HM 360; Microm Laborgeräte GmbH, Walldorf, Germany), mounted on glass slides and stained with haematoxylin and eosin.

### DNA extraction and determination of the viral load

DNA was extracted from 25 mg of liver and pancreas tissue from four birds of groups L1 and L2 at 4, 7 and 10 dpi, and from four and five birds at 4 and 5–7 dpi, respectively, of groups B1 and B2. For this, the DNeasy Blood and Tissue Kit (Qiagen, Vienna, Austria) was used following the manufacturer’s instructions. The extracted DNA was stored at −20 °C until use. A SYBR Green based real-time PCR with primers annealing within the highly conserved 52 K region was performed to determine the viral load, as described by Günes et al. [12]. The real-time PCR was performed on a Rotor-Gene Q thermal cycler (Qiagen, Hilden, Germany), using the double-stranded DNA-binding dye method with a Rotor-Gene SYBR Green PCR kit (Qiagen). During the annealing/extension step data were collected being further analysed in the Rotor-Gene Q software 1.7 (Qiagen). Standard curves were obtained by preparing 10-fold serial dilutions of a linearized plasmid containing the partial 52 K gene of a FAdV-D strain (SR49) and were run two times in duplicate. During sample preparation and real-time PCR run, negative extraction control and no template control (NTC) were included to monitor possible contaminations. The number of viral genome copies per reaction was calculated by comparing threshold cycle (C_T_) values of the investigated samples with the standard curves. An assessment of the specificity of the real-time PCR products was accomplished by analysing the melting curve together with the separation of the amplification products by electrophoresis.

### Statistical analysis

A Shapiro–Wilk test was performed together with a visual inspection of histograms, normal Q–Q plots and an assessment of skewness and kurtosis z-values to confirm the normal distribution assumptions of the data within each group. Viral load data were log transformed to meet the normality assumptions. Survival curves were estimated by the Kaplan–Meier method, in which routinely killed birds were censored. A pairwise comparison by the log-rank test was performed to investigate the significance of differences in survival rates. Survival rate data were presented in terms of cumulative mortality (1 minus the survival rate). An unpaired *t* test was used to compare the body weight and the clinical chemistry results from each infected group with their respective control group at each time point. Statistical differences regarding the viral load in liver and pancreas between broilers and layers infected with the same strain at each time point were investigated by a one-way ANOVA succeeded by pairwise comparisons using the Gabriel post hoc test. In all cases, significant differences were assumed when *P* < 0.05. Data were analysed with the statistical software package SPSS Version 22 (IBM SPSS Statistics; IBM Corporation, Armonk, New York, USA).

## Results

### Clinical signs, mortality and body weight

**Figure 1:**
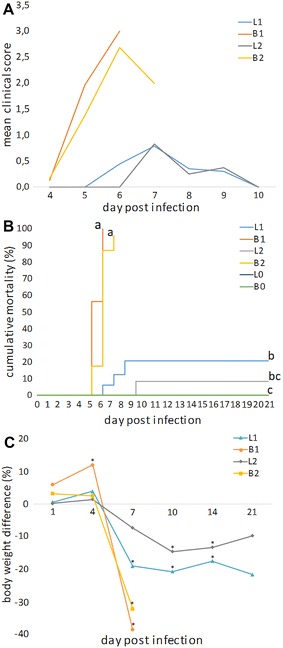
**Mean clinical score, cumulative mortality and mean body weight difference. A** Infected birds belonging to groups L1, B1, L2 and B2 were individually scored based on the following clinical signs: 0—active with no clinical signs; 1—slightly weak with dropped wings; 2—depressed with swollen crops; 3—weak, apathetic, with ruffled feathers and reluctant to move; 4—apathetic, unable to move or stand, breathing intensely with eyes closed. An average of each group’s clinical score was calculated at each time point. All clinical signs were observed between 4 and 9 dpi. No clinical signs were observed in the control birds. **B** Mortality rates (%), recorded in groups L1, B1, L2, B2, L0 and B0 throughout the animal experiment. Mortality curves with different lowercase letters are significantly different (*P* < 0.05). **C** Mean differences in body weight (%) of infected birds belonging to groups L1, B1, L2 and B2 in comparison to the respective control group (L0 or B0), at 1, 4, 7, 10, 14 and 21 dpi. Values of groups B1 and B2 at 7 dpi correspond to pooled birds that were euthanized and sampled between 5 and 7 dpi due to poor condition. Asterisks indicate statistical significant difference (*P* < 0.05). From 7 dpi onwards there were no SPF broilers alive in groups B1 and B2.

Specific pathogen-free broiler chickens from groups B1 and B2 showed severe clinical signs starting at 4 dpi, with high clinical scores reached at 6 dpi (Figure 1A). The condition of the SPF broilers downgraded very quickly and significant mortalities of 100 and 96% were recorded between 5 and 6 dpi in group B1 and 5–7 dpi in group B2, respectively (Figure 1B). Moreover, the body weight of the inoculated SPF broilers was found to be significantly lower at 5–7 dpi compared with the body weight of SPF broilers from the control group (B0) (Figure 1C).

Specific pathogen-free layer chickens showed milder clinical signs in comparison to the broilers, from 6 to 9 dpi, and reached a peak at 7 dpi in both groups L1 and L2 (Figure 1A). In these groups, mortalities of 20 and 8% were recorded between 6 and 8 or at 9 dpi, respectively, being significantly lower when compared to both groups B1 and B2 (Figure 1B). Furthermore, a significantly lower body weight was already observed in birds from group L1 at 7 dpi and in birds from both groups L1 and L2 at 10 and 14 dpi when compared to the control group (L0) (Figure 1C).

No clinical signs and mortality were recorded in the control groups (L0 and B0).

### Gross pathology

In all SPF broiler chickens from groups B1 and B2 killed at 4 dpi, swollen livers were the most prominent finding during necropsy. Additionally, small necrotic foci were present in the liver of one bird from group B2. At this time point, no other lesions were recorded. Furthermore, no pathomorphological lesions were observed in SPF layers. However, all dead and killed birds of the inoculated groups between 5 and 9 dpi, regardless of host and virus strain, presented swollen marble-like livers with a colour ranging from yellow to brown. Moreover, swollen kidneys were observed during the necropsy of two SPF layer chickens (one found dead and one killed) from group L1 at 7 dpi, whereas in SPF broilers no lesions were found in kidneys.

No gross lesions were observed in other organs. Furthermore, no macroscopical changes were present in organs from birds of the control groups (L0 and B0).

### Histopathology

Although microscopical lesions were already observed at 4 dpi, most severe histological changes in infected birds were recorded at 5–7 dpi, when large basophilic intranuclear inclusion bodies were observed in the hepatocytes and acinar cells of liver and pancreas, respectively, together with large areas of cellular degeneration and necrosis (Additional file 2). In coincidence with this, areas of lymphocyte infiltration were found in liver and pancreas of birds from groups L1 and L2. Lymphocyte depletion in bursa of Fabricius was observed in all birds from group B2 and in 1 and 2 birds from groups L1 and B1, respectively, whereas signs of atrophy of the bursa of Fabricius was observed in 1 bird each from groups B1, L2 and B2 at 5–7 dpi.

At 10 dpi, areas of lymphocyte infiltration in liver and pancreas were seen in all birds from groups L1 and L2, with very few birds presenting small areas of necrosis in liver and pancreas.

No histological changes were observed in the kidney. Furthermore, no microscopical lesions were present in organs from birds of the control groups (L0 and B0).

### Clinical chemistry

**Figure 2:**
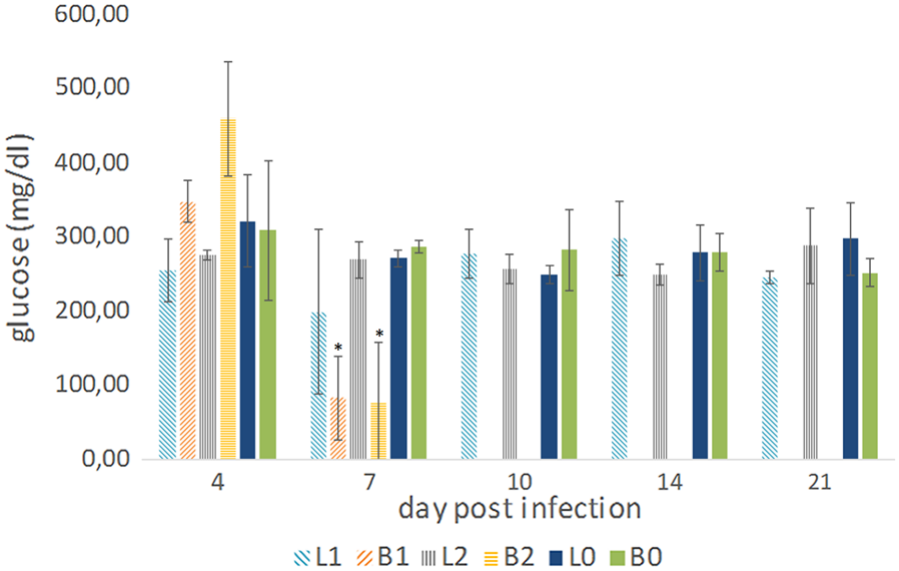
**Blood glucose concentration.** Means and standard deviations of blood glucose concentration values (mg/dL) of SPF layer and SPF broiler chickens from groups L1, B1, L2, B2, L0 and B0 at 4, 7, 10, 14 and 21 dpi. Values of groups B1 and B2 at 7 dpi correspond to pooled birds that were euthanized and sampled between 5 and 7 dpi due to poor condition. From 7 dpi onwards there were no SPF broilers alive in groups B1 and B2. All dead and killed birds at each investigated time point of each group (Table 1) were included. Asterisks indicate statistical significant difference when compared to the respective control group (L0 or B0) (*P* < 0.05).

The clinical chemistry results are presented in Table 2 and Figure 2. Total plasmatic protein was significantly changed in group B1 at 5–7 dpi and in group L2 at 14 dpi, being lower at the former and higher at the latter sampling point, when compared to their respective control group. The levels of plasmatic albumin were significantly lower at 5–7 dpi in all inoculated groups when compared to the levels observed in the respective control group.

**Table 2:** **Clinical chemistry analytes.** Means and standard deviations of total protein, albumin, AST, GLDH, bile acids, uric acid and lipase measured in the plasma of orally inoculated SPF layers and SPF broilers from groups L1, B1, L2, B2, L0 and B0 at 4, 7, 10, 14 and 21 days post infection (dpi).

Dpi^a^	Group	Total protein (g/dL)	Albumin (g/dL)	AST (U/L)	GLDH (U/L)	Bile acids (µmol/L)	Uric acid (mg/dL)	Lipase (U/L)
	L1	3.05 ± 0.16	1.41 ± 0.13	182.25 ± 17.10	9.27 ± 4.38	48.00 ± 15.25	6.73 ± 0.88^c^	5.75 ± 0.83
	B1	2.48 ± 0.27	1.09 ± 0.09	226.50 ± 18.98	8.85 ± 0.76	53.75 ± 4.57	15.48 ± 8.24	7.25 ± 0.43
4	L2	2.84 ± 0.22	1.35 ± 0.13	164.50 ± 3.32^c^	8.47 ± 2.44	41.75 ± 12.69	7.95 ± 0.95^c^	5.75 ± 1.09
	B2	1.93 ± 0.12	0.84 ± 0.06	256.75 ± 82.43	21.01 ± 21.18	51.00 ± 17.80	13.65 ± 5.57	6.00 ± 0.71
	L0	3.04 ± 0.16	1.28 ± 0.31	228.25 ± 35.72	8.51 ± 2.50	54.00 ± 6.98	12.48 ± 3.34	6.75 ± 1.78
	B0	2.16 ± 0.43	1.01 ± 0.20	211.67 ± 56.54	13.44 ± 19.43	49.67 ± 26.10	12.33 ± 3.84	9.75 ± 7.66
	L1	2.91 ± 0.44	0.99 ± 0.08^c^	1304.00 ± 1648.35	92.34 ± 148.87	49.75 ± 45.98	8.38 ± 4.00	78.50 ± 102.60
	B1^b^	1.96 ± 0.29^c^	0.64 ± 0.04^c^	2382.40 ± 817.10^c^	35.88 ± 8.43^c^	272.40 ± 129.75^c^	8.34 ± 3.42	36.25 ± 11.50^c^
7	L2	3.14 ± 0.11	1.13 ± 0.12^c^	425.25 ± 153.27^c^	6.21 ± 2.34	167.75 ± 68.27^c^	5.45 ± 1.06	35.33 ± 12.66^c^
	B2^b^	2.53 ± 0.37	0.84 ± 0.25^c^	4319.67 ± 2970.77^c^	268.59 ± 274.16	197.00 ± 158.42^c^	10.38 ± 2.55	271.40 ± 132.48^c^
	L0	3.23 ± 0.18	1.54 ± 0.1	166.50 ± 19.49	4.39 ± 2.07	24.00 ± 6.98	6.58 ± 0.77	5.75 ± 1.09
	B0	2.34 ± 0.22	1.25 ± 0.13	203 ± 8.09	13.19 ± 4.60	29.80 ± 7.53	8.60 ± 1.72	7.20 ± 0.98
	L1	3.15 ± 0.22	1.29 ± 0.12	237.00 ± 18.13^c^	30.60 ± 22.86	40.25 ± 12.50^c^	7.68 ± 2.31	7.00 ± 0.82^c^
10	L2	3.07 ± 0.36	1.29 ± 0.27	278.75 ± 129.96	9.80 ± 6.75	42.50 ± 8.39^c^	6.55 ± 1.16	20.25 ± 19.14
	L0	2.52 ± 0.42	0.70 ± 0.49	149.08 ± 26.61	5.52 ± 2.63	23.50 ± 9.20	6.87 ± 1.28	5.50 ± 0.50
	B0	2.32 ± 0.31	0.62 ± 0.44	182.17 ± 24.60	7.78 ± 4.50	30.83 ± 10.17	9.59 ± 2.25	8.80 ± 3.82
	L1	3.18 ± 0.20	1.33 ± 0.07	166.75 ± 6.99	4.17 ± 2.37	26.00 ± 9.83	6.53 ± 3.20	7.75 ± 3.03
14	L2	3.35 ± 0.21^c^	1.47 ± 0.04	225.00 ± 31.55^c^	66.35 ± 94.58	32.25 ± 2.63^c^	5.55 ± 1.50	5.33 ± 0.47
	L0	2.60 ± 0.42	0.84 ± 0.53	158.25 ± 29.93	5.10 ± 1.82	19.88 ± 5.96	6.78 ± 1.88	7.50 ± 1.12
	B0	2.46 ± 0.41	0.74 ± 0.47	162.31 ± 28.62	6.05 ± 4.54	21.92 ± 8.14	9.57 ± 1.42	5.80 ± 0.40
	L1	3.07 ± 0.30	1.27 ± 0.06	164.50 ± 4.95	4.61 ± 0.14	21.00 ± 4.24	5.10 ± 0.00	6.00 ± 0.00
21	L2	3.06 ± 0.28	1.43 ± 0.09	168.00 ± 21.63	3.12 ± 1.88	29.33 ± 7.57	5.47 ± 1.86	5.50 ± 0.50
	L0	3.22 ± 0.64	1.44 ± 0.40	182.00 ± 24.54	4.11 ± 1.46	26.25 ± 7.80	7.53 ± 1.69	6.50 ± 0.50
	B0	2.75 ± 0.17	1.23 ± 0.08	199.50 ± 18.61	10.29 ± 9.45	16.25 ± 11.35	7.81 ± 0.85	5.75 ± 0.97

From 10 dpi onwards there were no SPF broilers alive in groups B1 and B2. All dead and killed birds at each investigated time point of each group (Table 1) were included.

^a^Day post infection.

^b^Data from pooled birds, which were euthanized between 5 and 7 dpi due to poor condition.

^c^Statistical significant difference—*P* < 0.05 when compared to the respective control group (L1 and L2 compared to L0, and B1 and B2 compared to B0).

Two liver enzymes were measured in the plasma of the birds—AST and GLDH—and AST activity was significantly higher at 5–7 dpi in groups B1, L2, B2, in comparison to the respective control. At 7 dpi, high values of AST were also observed in group L1, however, a significant difference could not be found. At 10 and 14 dpi significantly higher values were recorded in groups L1 and L2, respectively, compared to the control group (L0). GLDH values were found to be very high at 5–7 dpi in groups L1, B1 and B2, at 10 dpi in group L1 and at 14 dpi in group L2. However, significant differences with the respective control were only found at 5–7 dpi in group B1.

Significant changes were observed in the levels of metabolites measured in the plasma. Bile acids plasmatic concentration was significantly increased at 5–7, 10 and 14 dpi in groups B1, L2 and B2, groups L1 and L2, and group L2, respectively. Plasmatic uric acid levels were significantly lower at 4 dpi in groups L1 and L2, in comparison to the control group L0.

Lipase activities were very high in all inoculated groups at 5–7 dpi, being significantly increased in groups B1, L2 and B2. At 10 dpi lipase activities were significantly and non-significantly increased in groups L1 and L2, respectively.

Levels of plasmatic glucose were non-significantly increased at 4 dpi in group B2 and significantly decreased at 5–7 dpi in groups B1 and B2, in comparison with the control group B0 (Figure 2).

### Viral load

**Figure 3:**
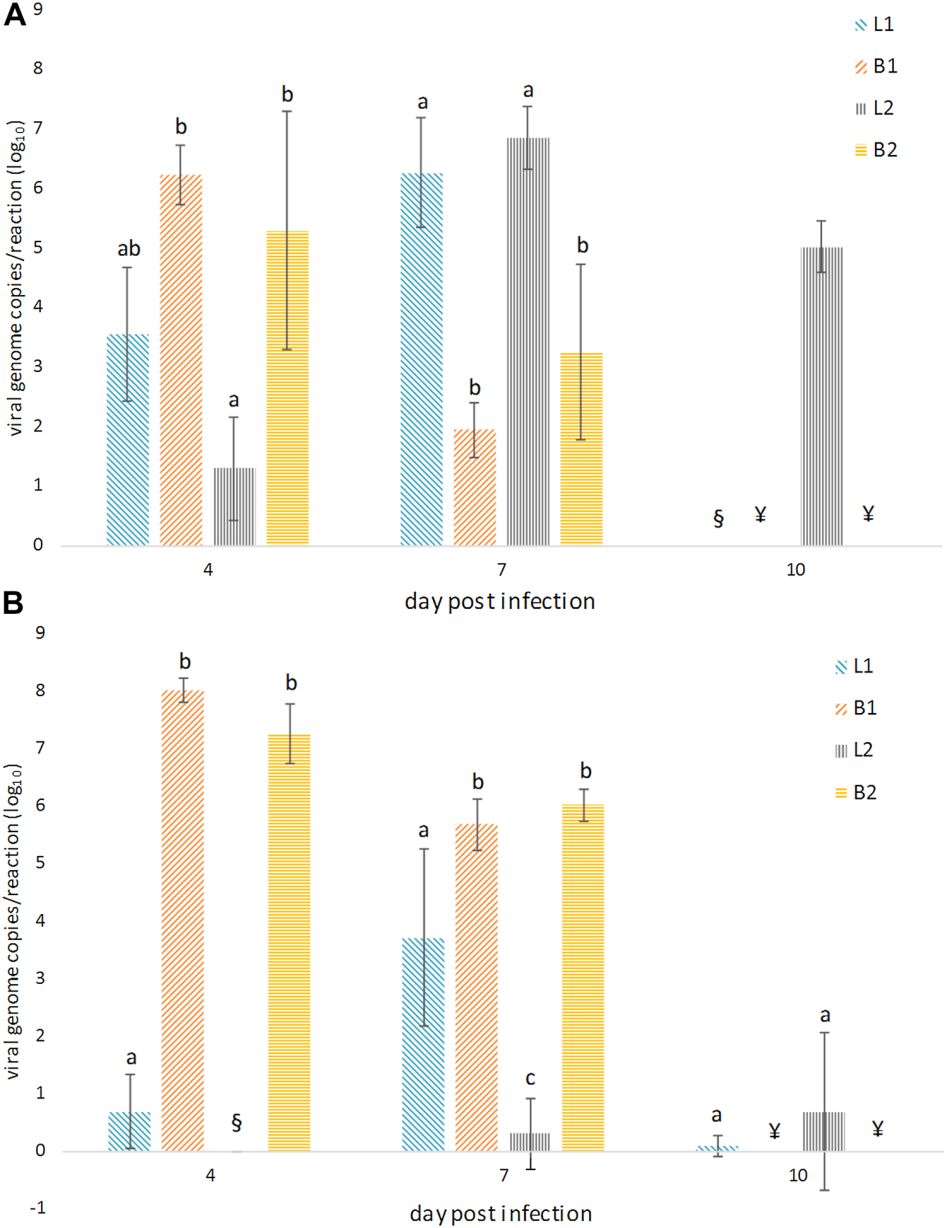
**Viral load in liver and pancreas.** Means and standard deviations of the viral genome copies per reaction (log_10_) in (**A**) liver and (**B**) pancreas of orally inoculated SPF layer and SPF broiler chickens from groups L1, B1, L2 and B2 at 4, 7 and 10 dpi quantified by real-time PCR. Values of groups B1 and B2 at 7 dpi correspond to pooled birds that were euthanized and sampled between 5 and 7 dpi due to poor condition. All dead and killed birds at each investigated time point from each group (Table 1) were included. Negative results are indicated with §. There were no SPF broilers alive at 10 dpi (¥). Mean values with different lowercase letters at each time point are significantly different (*P* < 0.05).

At 4 dpi the viral load in the liver of birds from both groups B1 and B2 was significantly higher in comparison to group L2 and non-significantly higher than in group L1 (Figure 3A). At 7 dpi, the viral load peaked in the liver of birds from groups L1 and L2 and the viral genome copies per reaction in these groups were significantly higher in comparison to groups B1 and B2 at 5–7 dpi.

In the pancreas, high loads of viral DNA were determined in both groups B1 and B2 at 4 and 5–7 dpi, being significantly higher compared with groups L1 and L2 (Figure 3B). Furthermore, the viral load in pancreas of birds from group L1 were higher in comparison to group L2 at 4 and 7 dpi.

## Discussion

In a recent study we used five FAdV field strains belonging to species FAdV-D and E to orally inoculate separate groups of day-old SPF white leghorn chickens and a panel of biomarkers based on clinical chemistry was established, which correlated with the pathogenicity of FAdV strains and the pathogenesis of IBH [9]. It was shown that pathogenic FAdV strains are capable to interfere with enzyme systems and metabolites concentration which are related to liver and pancreas functions.

In continuity with the aforementioned investigations, the aim of the present study was to compare and assess the influence of the genetic background of the host on the outcome of a FAdV infection considering the different metabolism and growth rate between meat-producing (SPF broilers) and egg-producing (SPF layers) chickens. For this, two previously tested FAdV-D and E field strains were chosen based on their pathogenicity in SPF layers [9]. Independent of the virus used for infection in the present investigation, significant mortalities approaching 100% together with severe clinical signs were recorded in the inoculated SPF broilers, differing substantially from the infection outcome in SPF layers. However, the recorded mortality in SPF layers was considerably lower when compared with the results which we previously reported, following the same inoculation procedure [9]. In the previous study, the occurrence of non-specific mortality in the first week of life together with a lower body weight of the control birds throughout the animal experiment, in comparison to the control SPF layers of the present study (data not shown), indicates a diminished quality and performance of the chicks, which may have influenced the outcome of the infection. Nonetheless, further investigations are needed to test this hypothesis.

Previous pathogenicity studies were mostly performed in layer-type chickens and mortalities of 30–50% were reported when birds were inoculated by natural routes with a similar dose of a virulent IBH strain [5, 9, 13–15], hence considerably lower than the mortalities recorded in the inoculated SPF broilers’ groups of the current study. Genetically different chickens were used earlier to investigate IBH in experimental studies [16–20]. However, in nearly all of these studies broilers of commercial origin were used, whose serological status regarding maternal antibodies against FAdVs was either positive or unknown [16, 18–20]. Exceptional to this, Cook [17] used different chicken breeds of SPF and non-SPF origin to study the influence of host, age and route of inoculation on the outcome of a FAdV infection. As a result, SPF Light Sussex chickens were highly susceptible to the infection in comparison to SPF Rhode Island Red chickens, with mortalities of 67 and 33% being recorded, respectively, following intraperitoneal inoculation at day-old. Nonetheless, no broiler chickens were used in the investigation. Different to this, Alvarado et al. [20] used broilers and layers of SPF origin together with broilers from vaccinated breeders to investigate the pathogenicity of a FAdV-E strain, and different mortalities in SPF broilers (40%) and SPF layers (20%) were reported, following subcutaneous infection at week-old. However, the study did not focus to elucidate the mechanisms of these differences in susceptibility, which would have been hampered by the fact that subcutaneous infection route was used. Thus, this is the first study to report a significant susceptibility of SPF broiler chickens following an oral infection with FAdV strains belonging to species FAdV-D and E, in direct comparison to SPF layers, and to unravel the background of this phenomenon.

Therefore, in the actual study, further investigations were carried out to elucidate the pathogenesis of IBH in general and the influence of the birds’ metabolism. Liver and pancreas are important target organs for FAdVs [9] and, therefore, the blood glucose concentration was determined in all birds throughout the study, as the glucose metabolism is a good indicator for liver and pancreas functions [21]. A recent investigation demonstrated that chickens from lines bred for high juvenile body weight have an impaired glucose homeostasis and a different pancreas physiology in comparison to chickens with a low juvenile body weight [22]. In the current investigation significantly lower blood glucose concentrations were recorded in both inoculated SPF broilers’ groups during the peak of infection, in comparison to the control group, corresponding to a hypoglycaemic status according to previously established reference intervals [21, 23]. It seems that IBH, to which broilers are highly susceptible, evolves to a metabolic disorder. Furthermore, significant changes in clinical chemistry analytes measured in the plasma of infected birds confirmed tissue damage and functional impairment of both liver and pancreas, which were further validated by histopathological studies. In a field investigation, Goodwin et al. [24] reported the presence of adenoviral inclusion bodies in liver, pancreas and small intestine of hypoglycaemic broiler chicks from a flock suffering from spiking mortality, to which the outcome of the present experimental study parallels. Glucose together with sodium bicarbonate and calcium were once effective as a supportive treatment during an IBH outbreak, in which broiler chickens were suffering from hypoglycaemia, metabolic acidosis and hypocalcaemia [25], highlighting the potential of FAdV infections to unfold in a metabolic impairment. It can be hypothesized that the blood glucose of the infected birds would decrease due to apathy and anorexia together with malabsorption related to the ongoing pancreatitis caused by the infection. However, during short-term fasting periods, the blood glucose concentration in granivorous birds is maintained by hepatic glycogenolysis, in which glucagon—a hormone produced by pancreatic alpha-cells—plays an important role as a regulator [21]. Therefore, severe lesions in liver and/or pancreas can contribute towards disturbances in the glucose homeostasis. Recently, it was demonstrated that birds with extensive lesions in pancreas due to an experimental infection with low-pathogenic avian influenza viruses (LPAIVs) experienced hyperglycaemia and not hypoglycaemia as observed in our study [26, 27]. In comparison, FAdV induced IBH severely affects not only the pancreas but also the liver of birds, preventing compensation mechanisms to occur. Nevertheless, further studies would be of interest to fully address this issue.

In addition to hepatic and pancreatic changes, lesions in kidneys have been described throughout the years as a consequence of experimental FAdV infections [9, 15, 28–30]. Kidneys are mostly responsible for removing uric acid from the blood and, therefore, concentrations greater than 13 mg/dL are suggestive for impaired renal function [21]. This was never the case in the present study and, in fact, plasmatic uric acid concentration was significantly lower only in the infected SPF layers’ groups at 4 dpi when compared with the control group. This can rather be interpreted as a consequence of liver instead of kidney damage, since the liver is highly responsible for uric acid production [21].Therefore, the findings of the present study do not provide evidence of kidney function impairment due to FAdV infection, confirming our previously published data [9]. In agreement with this, no lesions were observed in the kidneys during the histological investigation, despite of swollen kidneys noticed during necropsy in two birds. Although glomerulonephritis has been suggested as the underlying cause for the macroscopical changes in kidneys of broilers suffering from IBH in the field [31], a direct connection between FAdVs and glomerulonephritis has not been demonstrated, so far, in experimentally infected birds. Assessing the glomerular size and cellularity in kidneys of experimentally FAdV infected birds by histomorphometric studies would be of interest to test this hypothesis.

In our previous studies we were aiming to establish a link between viral load in target organs with histopathological and macroscopical lesions, clinical chemistry and clinical signs, in chickens experimentally infected with FAdVs, although different diseases were investigated and not all parameters were investigated at the same time [9, 32]. In the present study, very high loads of viral DNA were seen already at 4 dpi in both liver and pancreas of infected SPF broilers, preceding the onset of severe clinical signs and high mortality together with severe changes in clinical chemistry and histopathological lesions. Thus, these findings provide evidence that FAdVs replicate faster in both liver and pancreas of broilers in comparison to layers, harmonizing with the outcome of the infection. Furthermore, independent of the virus, much higher viral loads were determined in the pancreas of broilers in comparison to livers throughout the experiment, something not observed in layers. This highlights the importance of the pancreas as a target organ for FAdVs with its crucial function in context of the noticed metabolic derangement.

In conclusion, this is the first study to report a significant difference in susceptibility of SPF broiler chickens to an oral infection with FAdV strains belonging to species FAdV-D and E in direct comparison to SPF layers, underlining the importance of the genetic background of the host on the outcome of a FAdV infection. Furthermore, during the peak of infection SPF broilers suffered from hypoglycaemia, in which the severe lesions in liver and pancreas seemed to play an important role. Therefore, we propose that FAdV infections, to which broiler chickens are very susceptible, can lead to metabolic disorders. Nevertheless, further studies are needed to better understand the pathogenesis of IBH in broilers and the involvement of the liver and pancreas in metabolic derangements by FAdVs.

## Additional files

10.1186/s13567-016-0350-0 **Kits and methods used to investigate the clinical chemistry analytes in the plasma of birds.** Total protein, albumin, aspartate aminotransferase (AST), glutamate dehydrogenase (GLDH), bile acids, uric acid and lipase were investigated in the plasma of birds at different time points by a fully selective clinical chemistry analyzer (Cobas 501c^®^, Roche Diagnostics, Vienna, Austria).
10.1186/s13567-016-0350-0 **Histopathological findings in infected birds at different time points.** Histopathological lesions recorded in liver, pancreas and bursa of Fabricius of infected birds from groups L1, B1, L2 and B2 at 4, 7 and 10 dpi. No histopathological changes were observed in the kidney. Furthermore, no microscopical lesions were present in organs from birds of the control groups.

## Abbreviations

AGE: adenoviral gizzard erosion
AST: aspartate aminotransferase
CEL: chicken embryo liver
C_T_: threshold cycle
FAdV: fowl aviadenovirus
GLDH: glutamate dehydrogenase
HHS: hydropericardium hepatitis syndrome
IBH: inclusion body hepatitis
NTC: negative template control
PCR: polymerase chain reaction
SPF: specific-pathogen-free
TCID_50_: median tissue culture infective dose
