# Supplementary material for: The outcome of experimentally induced inclusion body hepatitis (IBH) by fowl aviadenoviruses (FAdVs) is crucially influenced by the genetic background of the host
Source: Vet Res. 2016 Jun 29;47:69. doi: 10.1186/s13567-016-0350-0 (PMC4928300; doi:10.1186/s13567-016-0350-0)
Supplement: Supplementary file 1 — 10.1186/s13567-016-0350-0 Kits and methods used to investigate the clinical chemistry analytes in the plasma of birds. Total protein, albumin, aspartate aminotransferase (AST), glutamate dehydrogenase (GLDH), bile acids, uric acid and lipase were investigated in the plasma of birds at different time points by a fully selective clinical chemistry analyzer (Cobas 501c®, Roche Diagnostics, Vienna, Austria). [file 13567_2016_350_MOESM1_ESM.docx]

| **Analyte** | **Test-kit** | **Principle** |
| --- | --- | --- |
| total protein | Total Protein/TP2 ™, Roche Diagnostics | Colorimetric, Biuret reaction |
| albumin | Albumin/ALB2™, Roche Diagnostics | Colorimetric bromcresol-green |
| aspartate-aminotransferase (AST) | Aspartate Aminotransferase/AST ™, Roche Diagnostics | IFCC kinetic method with pyridoxalphosphate activation |
| glutamate-dehydrogenase (GLDH) | Glutamatdehydrogenase/  GLDH ™, Roche Diagnostics | Kinetic, UV-Test; substrate: α-ketoglutarate |
| bile acids | Bile Acids/TBA™, Randox | Enzymatic (3-α-Hydroxisteroid-dehydrogenase), colorimetric assay |
| uric acid | Uric Acid/UA 2™, Roche Diagnostics | Enzymatic (Uricase) colorimetric assay, |
| lipase | Lipase/LIPC | Kinetic-colorimetric test; substrate: 1,2-O-dilaryl-rac-glycero3-glutaric acid – ester (6-methylresorufin)-ester |
