# Supplementary material for: The outcome of experimentally induced inclusion body hepatitis (IBH) by fowl aviadenoviruses (FAdVs) is crucially influenced by the genetic background of the host
Source: Vet Res. 2016 Jun 29;47:69. doi: 10.1186/s13567-016-0350-0 (PMC4928300; doi:10.1186/s13567-016-0350-0)
Supplement: Supplementary file 2 — 10.1186/s13567-016-0350-0 Histopathological findings in infected birds at different time points. Histopathological lesions recorded in liver, pancreas and bursa of Fabricius of infected birds from groups L1, B1, L2 and B2 at 4, 7 and 10 dpi. No histopathological changes were observed in the kidney. Furthermore, no microscopical lesions were present in organs from birds of the control groups. [file 13567_2016_350_MOESM2_ESM.docx]

|  |  | **liver** | | |  | **pancreas** | | |  | **bursa of Fabricius** | |
| --- | --- | --- | --- | --- | --- | --- | --- | --- | --- | --- | --- |
| **dpi^a^** | **group** | **inclusion bodies** | **necrosis** | **lymphoid infiltration** |  | **inclusion bodies** | **necrosis** | **lymphoid infiltration** |  | **lymphoid depletion** | **atrophy** |
| 4 | L1 | 0/4^b^ | 1/4 | 2/4 |  | 0/4 | 0/4 | 2/4 |  | 0/4 | 0/4 |
|  | B1 | 0/4 | 1/4 | 1/4 |  | 1/4 | 0/4 | 0/4 |  | 0/4 | 0/4 |
|  | L2 | 0/4 | 1/4 | 1/4 |  | 0/4 | 0/4 | 2/4 |  | 0/4 | 0/4 |
|  | B2 | 2/4 | 0/4 | 2/4 |  | 0/4 | 0/4 | 0/4 |  | 0/4 | 0/4 |
|  |  |  |  |  |  |  |  |  |  |  |  |
| 7 | L1 | 4/4 | 2/4 | 4/4 |  | 4/4 | 2/4 | 4/4 |  | 1/4 | 0/4 |
|  | B1^c^ | 5/5 | 5/5 | 0/5 |  | 3/5 | 4/5 | 0/5 |  | 2/5 | 1/5 |
|  | L2 | 4/4 | 4/4 | 4/4 |  | 4/4 | 4/4 | 2/4 |  | 0/4 | 1/4 |
|  | B2^c^ | 5/5 | 5/5 | 0/5 |  | 5/5 | 5/5 | 0/5 |  | 5/5 | 1/5 |
|  |  |  |  |  |  |  |  |  |  |  |  |
| 10 | L1 | 0/4 | 0/4 | 4/4 |  | 0/4 | 2/4 | 4/4 |  | 0/4 | 0/4 |
|  | B1 | -^d^ | - | - |  | - | - | - |  | - | - |
|  | L2 | 0/4 | 1/4 | 4/4 |  | 0/4 | 1/4 | 4/4 |  | 0/4 | 0/4 |
|  | B2 | - | - | - |  | - | - | - |  | - | - |

^a^ Day post infection.

^b^ No. birds positive/no. birds examined.

^c^ Birds euthanized due to poor condition between 5-7 dpi.

^d^ Not applicable.
